# Biomarkers of mitochondrial dynamics in idiopathic pulmonary fibrosis: Identification and validation through transcriptomic and single-cell analyses

**DOI:** 10.1371/journal.pone.0347845

**Published:** 2026-04-23

**Authors:** Yongbin Wang, Peng Cheng, Zixiang Meng, Ying Zhang, Yan Li, Feifei Feng

**Affiliations:** 1 The Second Qilu Hospital of Shandong University, Jinan, China; 2 Shandong University, Cheeloo College of Medicine, Second Clinical College, Jinan, China; Chung Shan Medical University, TAIWAN

## Abstract

**Background:**

Idiopathic pulmonary fibrosis (IPF) is a progressive fibrotic lung disease that has increasingly been associated with dysregulated mitochondrial quality control and dynamics. However, the molecular mechanisms underlying these alterations remain incompletely understood. This study aimed to systematically identify and validate candidate biomarkers related to mitochondrial dynamics in IPF and to characterize their cell-type specificity and putative regulatory relationships.

**Methods:**

We integrated bulk transcriptomic datasets from the Gene Expression Omnibus (GEO), single-cell RNA sequencing (scRNA-seq) data, and literature-derived mitochondrial dynamics gene sets. Candidate genes were identified through differential expression analysis and consensus clustering, followed by functional enrichment and protein–protein interaction (PPI) network analyses. A total of 101 machine-learning model combinations—including random forest, LASSO, and support vector machine—were constructed to select optimal feature genes. Diagnostic performance was assessed using receiver operating characteristic (ROC) analysis and further evaluated with artificial neural network (ANN) modeling. Additional analyses included chromosomal localization, immune infiltration profiling, multilayer regulatory network construction (transcription factors, lncRNAs, circRNAs), molecular docking prediction, and single-cell expression and pseudotime trajectory analysis. Key biomarkers were further evaluated by RT-qPCR in an independent clinical cohort.

**Results:**

Integrated multi-omics and machine-learning analyses identified *CD247*, *IL7R*, and *RETN* as candidate biomarkers related to mitochondrial dynamics-associated pathways in IPF. Across independent transcriptomic datasets, *RETN* was upregulated, whereas *CD247* and *IL7R* were downregulated, and each showed diagnostic value (single-gene AUC > 0.7). The ANN model based on these genes achieved encouraging discriminative performance (training AUC = 0.91; validation AUC = 0.82), and expression differences were confirmed by RT-qPCR in a modest independent cohort. Enrichment analyses indicated convergence on spliceosome-related pathways, and regulatory-network analysis highlighted interactions involving transcription factors and non-coding RNAs, including circRNA *CDR1as*. Molecular docking suggested putative interactions with selected compounds. Single-cell analyses suggested that dysregulation was most evident in monocyte-associated compartments in one publicly available scRNA-seq dataset, and pseudotime analysis indicated dynamic expression patterns, with early transient increases in *CD247* and *IL7R* and progressive elevation of *RETN*.

**Conclusion:**

Through multi-omics integration and machine-learning approaches, we identified and preliminarily validated *CD247*, *IL7R*, and *RETN* as candidate biomarkers related to mitochondrial dynamics–associated pathways in IPF. These findings provide transcriptomic and cell-type–specific evidence suggesting potential immune–mitochondrial associations in IPF and may inform future biomarker validation and mechanistic hypothesis generation.

## Introduction

Idiopathic pulmonary fibrosis (IPF) is a chronic, progressive, and frequently fatal interstitial lung disease characterized by relentless parenchymal thickening and fibrosis of unknown etiology. Patients experience irreversible loss of lung function, worsening dyspnea, and ultimately respiratory failure; median survival remains only 3–5 years after diagnosis [1,2]. As the most common idiopathic interstitial pneumonia (IIP), IPF is histopathologically defined by the usual interstitial pneumonia (UIP) pattern [1]. Incidence and prevalence are increasing worldwide and disproportionately affect older adults, imposing substantial burdens on patients, families, and healthcare systems [3].

Despite extensive investigation, disease-modifying options remain limited. The antifibrotic agents pirfenidone and nintedanib can slow physiological decline but do not reverse established fibrosis and are typically administered without consideration of inter-patient heterogeneity [3,4]. Lung transplantation is the only curative therapy, yet its use is constrained by organ availability and stringent eligibility criteria [5]. Although clinical characterization has advanced, the molecular and cellular mechanisms that drive IPF pathogenesis remain incompletely defined. A dysregulated wound-healing response to recurrent alveolar epithelial injury is considered central to disease progression [6], but the precise pathways governing this process are still unclear.

A major challenge to improving IPF management is the lack of reliable biomarkers that enable early diagnosis, predict disease trajectory, and support personalized therapy. Recent studies underscore the need for novel biomarkers to improve risk stratification and treatment outcomes. Among implicated mechanisms, mitochondrial dysfunction has increasingly been recognized as a contributor to IPF pathobiology. Mitochondria are essential for cellular homeostasis, and their dysregulation has been linked to alveolar epithelial apoptosis, fibroblast activation, and metabolic reprogramming—hallmarks of pulmonary fibrosis [7,8]. Mitochondrial dynamics—the tightly regulated balance between fission and fusion—critically influence cell-fate decisions and tissue remodeling, yet their specific roles in IPF remain incompletely understood. Disruption of these processes may contribute to IPF pathogenesis and may also have potential relevance for biomarker discovery and future therapeutic investigation. Emerging evidence further suggests that mitochondrial dysfunction, particularly its crosstalk with alveolar macrophages, amplifies profibrotic signaling in IPF [9].

Despite growing recognition of mitochondrial involvement, the identification and validation of mitochondrial dynamics–related biomarkers remain incomplete. Current studies indicate that mitochondrial dysregulation in alveolar macrophages and epithelial cells may promote fibrosis, yet the specific molecular mechanisms and key regulatory genes are largely undefined [10–12]. Given the biological relevance of mitochondrial dynamics to cellular stress responses and immune regulation, further investigation of mitochondrial dynamics–related genes (MRGs) may help clarify their potential molecular functions and regulatory networks in IPF. Importantly, our focus on MRGs is based on a hypothesis-driven and biologically grounded framework, rather than on the assumption that these genes are already well established as dysregulated in IPF. For example, examining the expression and functional roles of core fission and fusion regulators—dynamin-related protein 1 (Drp1) and optic atrophy 1 (OPA1)—which are known to regulate immune activation and cellular stress responses, may help clarify whether they contribute to IPF-related immune dysregulation and may identify candidate targets for future investigation [13,14].

To bridge these knowledge gaps, this study integrates bioinformatics with machine-learning approaches to systematically interrogate mitochondrial dynamics–related genes and identify candidate biomarkers in IPF. We aimed to characterize their biological functions, define putative regulatory networks, and examine their expression patterns at single-cell resolution to identify major cell populations associated with disease-related changes. Ultimately, this work seeks to provide a transcriptomic and cell-type–resolved framework for biomarker-guided diagnostics and for generating mechanistic hypotheses for future experimental testing in IPF.

## Materials and methods

### Data collection

Bulk transcriptomic and single-cell RNA-sequencing (scRNA-seq) datasets were retrieved from the Gene Expression Omnibus (GEO). The training dataset was GSE93606, which included peripheral whole-blood samples from 57 patients with idiopathic pulmonary fibrosis (IPF) and 20 healthy controls [15]. The validation dataset was GSE28042, comprising peripheral blood mononuclear cell (PBMC) samples from 75 patients with IPF and 19 healthy controls [16]. Single-cell RNA-seq data were obtained from GSE233844, which contained PBMCs from 13 patients with IPF and 13 healthy controls [17]. In addition, a curated list of 23 mitochondrial dynamics-related genes (MRGs) was compiled based on published literature [18].

These GEO datasets were selected for their relevance to IPF and their suitability for the study design. GSE93606 served as the training cohort for differential expression and machine-learning analyses, whereas GSE28042 was used as an independent validation cohort to assess biomarker reproducibility across platforms. GSE233844 was included for single-cell analyses because it provided annotated PBMC scRNA-seq data from patients with IPF and controls, enabling immune cell-specific expression profiling and trajectory analysis. All public datasets were de-identified, and the original studies had obtained institutional ethics approval and informed consent.

### Mitochondrial dynamics–related gene (MRG) curation

Mitochondrial dynamics–related genes (MRGs) were curated using a literature-based strategy. Genes were included if they were functionally annotated as regulators of mitochondrial fission, fusion, or quality control, supported by prior experimental evidence, or previously implicated in pulmonary fibrosis or lung immune dysregulation [7,12,18]. Candidate genes were identified through a review of published studies on mitochondrial dynamics and fibrosis-related mechanisms, supplemented by functional annotation from GeneCards. Core regulators of mitochondrial fission (e.g., DNM1L/DRP1) and fusion (e.g., MFN1, MFN2, OPA1) were prioritized. Using these criteria, a final set of 23 MRGs was assembled, representing key biological processes governing mitochondrial dynamics.

### Identification of candidate genes

Differentially expressed genes (DEGs) between IPF and control samples in GSE93606 were identified using the limma package [19], with thresholds of *p* < 0.05 and |log₂ fold change| > 0.5. To characterize MRG-associated expression patterns, IPF samples in GSE93606 were grouped using consensus clustering based on MRG expression profiles. DEGs between MRG-defined clusters were identified using the same criteria. Candidate genes were defined as the intersection between IPF-associated DEGs and MRG cluster–associated DEGs.

### Functional enrichment and protein–protein interaction (PPI) analysis

Functional enrichment analysis of candidate genes was performed using Gene Ontology (GO) and Kyoto Encyclopedia of Genes and Genomes (KEGG) pathway analyses implemented in the clusterProfiler package [20]. Statistical significance was determined using the Benjamini–Hochberg (BH) correction for multiple testing, with adjusted p < 0.05 considered significant. For each biomarker, the top three significantly enriched pathways were retained for interpretation and visualization. Protein–protein interaction (PPI) networks were constructed using the STRING database with a minimum interaction confidence score of 0.4. Densely connected modules were identified using the MCODE plugin in Cytoscape [21] with the following parameters: K-core = 2, degree cutoff = 2, node score cutoff = 0.2, and max depth = 100. Genes from the highest-scoring module were selected for downstream analyses and considered candidate biomarkers.

### Machine-learning–based biomarker screening

To robustly identify diagnostic biomarkers, we constructed 101 composite machine-learning models integrating commonly applied algorithms, including random forest (RF), least absolute shrinkage and selection operator (LASSO), ridge regression, elastic net (Enet), support vector machine (SVM), generalized linear model boosting (glmBoost), stepwise generalized linear modeling (Stepglm), linear discriminant analysis (LDA), gradient boosting machine (GBM), extreme gradient boosting (XGBoost), and Naïve Bayes. All models were trained in the GSE93606 cohort and independently evaluated in the external validation cohort GSE28042. To balance model parsimony and predictive performance, composite models were constrained to include 3–7 variables. During training, hyperparameters were optimized through internal cross-validation to reduce overfitting and enhance robustness; elastic net–based models selected optimal regularization parameters via 10-fold cross-validation, boosting algorithms determined optimal tree numbers or iterations through cross-validation, and other learners applied analogous internal validation strategies for parameter tuning. Model performance in the training cohort was assessed using 10-fold cross-validation, with mean area under the receiver-operating-characteristic curve (AUC) serving as the primary selection criterion. The model achieving the highest and most stable AUC under the predefined variable constraint was selected as the optimal classifier. Classification performance was further evaluated using confusion-matrix–derived metrics, including sensitivity, specificity, precision, and F1 score. After internal optimization and feature selection in GSE93606, all candidate models were evaluated once in the independent GSE28042 cohort to assess generalizability. Single-gene diagnostic performance was subsequently examined using ROC analysis (pROC v1.18.0), and genes demonstrating consistent expression directionality and AUC > 0.7 in both cohorts were designated as candidate biomarkers.

### Artificial neural network modeling

Artificial neural network (ANN) models were constructed to evaluate the predictive performance of the identified biomarkers. In the training cohort (GSE93606), biomarker expression values were min–max scaled to the range [0,1] prior to model development. ANNs were implemented using the NeuralNetTools (v1.5.3) and neuralnet (v1.44.2) packages in R. A feedforward network with a single hidden layer was adopted, consisting of three input nodes corresponding to the three biomarkers, three hidden neurons, and two output nodes for binary classification (IPF vs. control). The cross-entropy error function (err.fct = “ce”) was used as the loss function, with a nonlinear activation function (linear.output = FALSE). Model training employed the default resilient backpropagation algorithm with weight backtracking, and convergence was determined using a threshold of 0.5 without additional regularization. Model performance was assessed using confusion matrices and receiver operating characteristic (ROC) curve analysis (pROC v1.18.0), with the area under the ROC curve (AUC) used to quantify discriminative ability; AUC > 0.7 was considered indicative of acceptable performance.

### Chromosomal localization and correlation analysis

Chromosomal locations of biomarkers were obtained from the Ensembl genome browser and visualized accordingly. Pairwise correlations among biomarkers were assessed using Spearman correlation analysis in GSE93606, with |r| > 0.3 and *p* < 0.05 considered statistically significant.

### Functional association and gene set enrichment analyses

Biomarker-associated functional networks were constructed using GeneMANIA to generate biomarker-centered interaction networks. Gene set enrichment analysis (GSEA) was performed by ranking genes in GSE93606 according to their Spearman correlation with each biomarker, using the curated KEGG gene set collection (c2.cp.kegg.v2023.1.Hs.symbols.gmt) as the reference set. GSEA was conducted with *clusterProfiler* (v4.2.2), applying Benjamini–Hochberg correction; pathways with adjusted p < 0.05 were considered significant, and the top three enriched pathways per biomarker were reported.

### Immune infiltration analysis

The relative abundance of 28 immune cell types in peripheral blood samples was estimated using single-sample gene set enrichment analysis (ssGSEA) based on established immune cell gene signatures [22]. Differences between IPF and control groups were assessed using the Wilcoxon rank-sum test (p < 0.05). Correlations between immune cell enrichment scores and biomarker expression levels were evaluated using Spearman correlation analysis.

The 28 immune cell types analyzed in this study were defined using curated gene signature sets from the Molecular Signatures Database (MSigDB), representing a standardized and widely used immune reference panel rather than an IPF-specific design. This panel includes major innate and adaptive immune cell populations and has been broadly applied in transcriptomic studies of chronic inflammatory and fibrotic diseases, including pulmonary fibrosis, making it suitable for evaluating immune alterations associated with IPF.

### Regulatory network construction

Potential transcription factors regulating biomarkers were identified using the JASPAR database. Candidate microRNAs were predicted using TargetScan and miRWalk, and overlapping predictions were retained. Long non-coding RNAs (lncRNAs) and circular RNAs (circRNAs) interacting with these microRNAs were identified using StarBase. Multilayer regulatory networks, including transcription factor–mRNA and noncoding RNA–mediated interactions, were visualized using Cytoscape.

### Drug prediction and molecular docking

Potential compounds associated with the identified biomarkers were predicted using the BATMAN-TCM and HERB databases, which curate bioactive molecules with reported relevance to immune and inflammatory processes. Three-dimensional structures of candidate compounds were retrieved from PubChem, and predicted protein structures for the biomarkers were obtained from AlphaFold. Molecular docking was performed using CB-Dock2. Predicted binding energy values were used as a relative measure to compare ligand–protein interactions, with more negative values indicating stronger predicted binding affinity.

### Single-cell RNA-seq analysis

scRNA-seq data from GSE233844 were processed using Seurat [23]. Cells expressing fewer than 200 genes or with excessive mitochondrial gene content (>10%) were excluded. Data normalization, identification of highly variable genes, dimensionality reduction, and clustering were performed using standard workflows. Cell types were annotated using the SingleR package by comparing cluster-level expression profiles against the Human Primary Cell Atlas (HPCA) reference dataset. HPCA contains transcriptomic signatures of major human immune and hematopoietic cell types and is widely used for PBMC-based single-cell RNA-seq annotation. This reference enabled the identification of monocytes, T cells, B cells, and other immune populations relevant to immune dysregulation in idiopathic pulmonary fibrosis. Biomarker expression patterns across cell types were visualized, and differential expression was assessed using the Wilcoxon test.

Cell clustering was performed using a resolution of 0.1 in Seurat to emphasize major immune cell populations rather than fine-grained subclusters. This low-resolution setting was chosen to align with the study objective of identifying major immune cell populations associated with candidate biomarkers related to mitochondrial dynamics-associated pathways, and to maintain consistency with bulk transcriptomic analyses derived from mixed PBMC populations. Higher resolutions (0.3, 0.5, and 1.0) were also explored and resulted in further subdivision of major lineages (e.g., T-cell and monocyte subsets); however, these alternative settings did not alter the overall biomarker expression patterns or the identification of monocytes as the predominant biomarker-associated cell population. Therefore, resolution 0.1 was retained for downstream analyses to balance biological interpretability and analytical parsimony. Accordingly, conclusions from this analysis were intended to prioritize broad immune-cell associations rather than definitive subcluster-level inference. In addition, because only a single public scRNA-seq dataset was analyzed, cell-type annotation and trajectory inference should be interpreted with caution and will require validation in independent datasets and with orthogonal approaches.

### Functional profiling, cell–cell communication, and trajectory analysis

Pathway enrichment across annotated cell types was assessed using ReactomeGSA [24]. Cell–cell communication was inferred using CellChat [25]. Pseudotime trajectory analysis was performed using Monocle [26] in the monocyte population to characterize dynamic gene expression changes.

### Experimental validation by reverse transcription quantitative real-time polymerase chain reaction (RT-qPCR)

Peripheral whole-blood samples were collected from 10 IPF patients and 10 healthy controls at the Department of Respiratory and Critical Care Medicine, Second Hospital of Shandong University. Total RNA was extracted and reverse-transcribed into cDNA, followed by quantitative real-time PCR (RT-qPCR) analysis. Relative mRNA expression levels were calculated using the 2^–ΔΔCt method, with GAPDH as the internal reference gene. Primer sequences are provided in Table 1.

**Table 1:** Primer sequences.

Primer	Sequence
RETN-F	CCCACCGAGAGGGATGAAAG
RETN-R	TGGCAGTGACATGTGGTCTC
CD247-F	GGCACAGTTGCCGATTACAGA
CD247-R	CTGCTGAACTTCACTCTCAGG
IL7R-F	CGGGAAGGAGCCAATGACTT
IL7R-R	ATACATTGCTGCCGGTTGGA
GAPDH-F	CGAAGGTGGAGTCAACGGATTT
GAPDH-R	ATGGGTGGAATCATATTGGAAC

### Statistical analysis

Statistical analyses were performed using R and GraphPad Prism. Two-tailed Student’s t tests were used for comparisons of RT-qPCR results, and the Wilcoxon rank-sum test was applied for group comparisons in transcriptomic analyses. Spearman’s correlation analysis was used to assess associations between biomarkers, immune cell signatures, and related phenotypes. Correlation coefficients with |r| > 0.3 and a two-sided p < 0.05 were considered indicative of moderate and potentially biologically meaningful associations, consistent with commonly adopted thresholds in transcriptomic and immune-related studies. A two-sided p < 0.05 was used to define statistical significance throughout the study. Unless otherwise specified, default software parameters were applied.

For analyses involving multiple comparisons, including functional enrichment (GO, KEGG, and GSEA) and correlation analyses, *p* values were adjusted using the Benjamini–Hochberg procedure to control the false discovery rate (FDR). An adjusted *p* value (FDR) < 0.05 was considered statistically significant.

### Ethics approval and consent to participate

This study was approved by the Scientific Research Ethics Committee of the Second Hospital of Shandong University (approval number KYLL2025593). All participants involved in the RT-qPCR validation provided written informed consent prior to enrollment. Public transcriptomic and single-cell RNA-seq datasets analyzed in this study were obtained from the Gene Expression Omnibus (GEO; GSE93606, GSE28042, and GSE233844). The original studies had obtained ethics approval and informed consent. As only de-identified public data were used for secondary analyses, no additional ethics approval was required for this component of the study.

## Results

### Identification and functional analysis of 152 candidate genes

**Fig 1:**
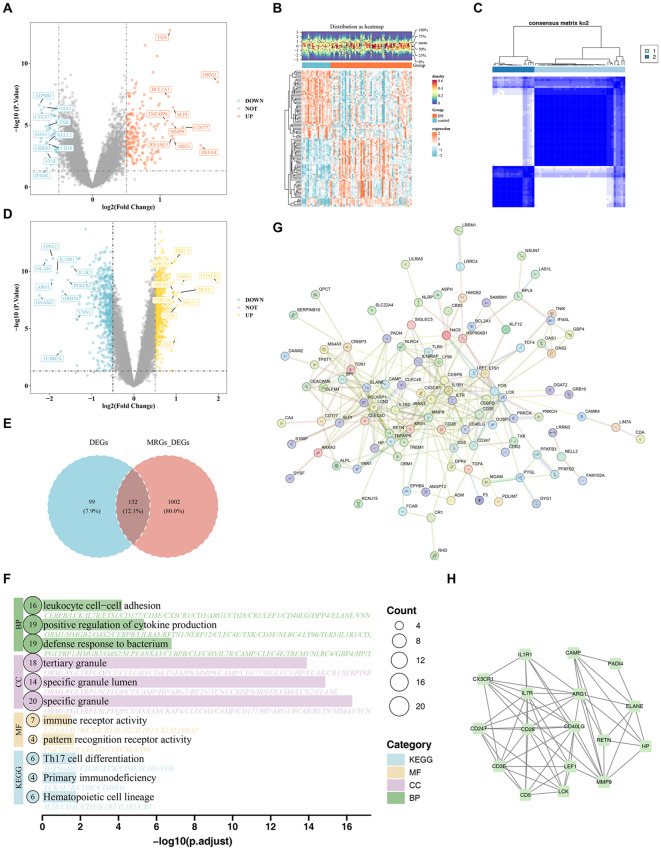
Identification and functional analysis of candidate genes associated with mitochondrial dynamics in IPF. (A) Volcano plot of differentially expressed genes between IPF and control groups. (B) Heatmap of differentially expressed genes. (C) Consensus clustering heatmap. (D) Volcano plot of differentially expressed genes between Cluster1 and Cluster2. (E) Venn diagram of candidate genes. (F) GO and KEGG enrichment analyses of candidate genes (the top three pathways). (G) PPI network of candidate genes. (H) Genes in the top-scoring module.

Differential expression analysis initially identified a total of 251 DEGs, including 193 upregulated and 58 downregulated genes in IPF samples relative to controls (p < 0.05 and |log2FC| > 0.5) (Fig 1A-B and S1 Table). To identify genes associated with MRGs, consensus clustering was performed in 57 IPF samples from the GSE93606 dataset. The optimal clustering solution was achieved at k = 2, yielding 39 samples in Cluster 1 and 18 samples in Cluster 2 (Fig 1C and S2 Table). Differential expression analysis between Cluster 1 and Cluster 2 identified 1,154 MRG-associated DEGs, including 659 upregulated and 495 downregulated genes in Cluster 1 (p < 0.05 and |log2FC| > 0.5) (Fig 1D). A total of 152 candidate genes were then identified by intersecting the IPF-associated DEGs with the MRG-associated DEGs (Fig 1E). These candidate genes were significantly enriched in 487 GO terms, including 415 biological processes (BPs) (e.g., defense response to bacterium), 34 cellular components (CCs) (e.g., specific granule), and 38 molecular functions (MFs) (e.g., pattern recognition receptor activity), as well as 29 KEGG pathways (e.g., hematopoietic cell lineage) (p < 0.05) (Fig 1F, S3 and S4 Tables). PPI network analysis of the candidate genes identified 104 nodes and 303 edges (Fig 1G). The highest-scoring module (score = 7.75) contained 17 candidate biomarkers: *ELANE, LEF1, IL1R1, LCK, PADI4, CAMP, CD3E, RETN, CD40LG, CD5, ARG1, MMP9, HP, CX3CR1, CD28, CD247,* and *IL7R* (Fig 1H). Among these, *MMP9, LCN2, FOS, ELANE, CD40LG,* and *PGLYRP1* interacted with more than 15 genes.

### *CD247, RETN* and *IL7R* as biomarkers

**Fig 2:**
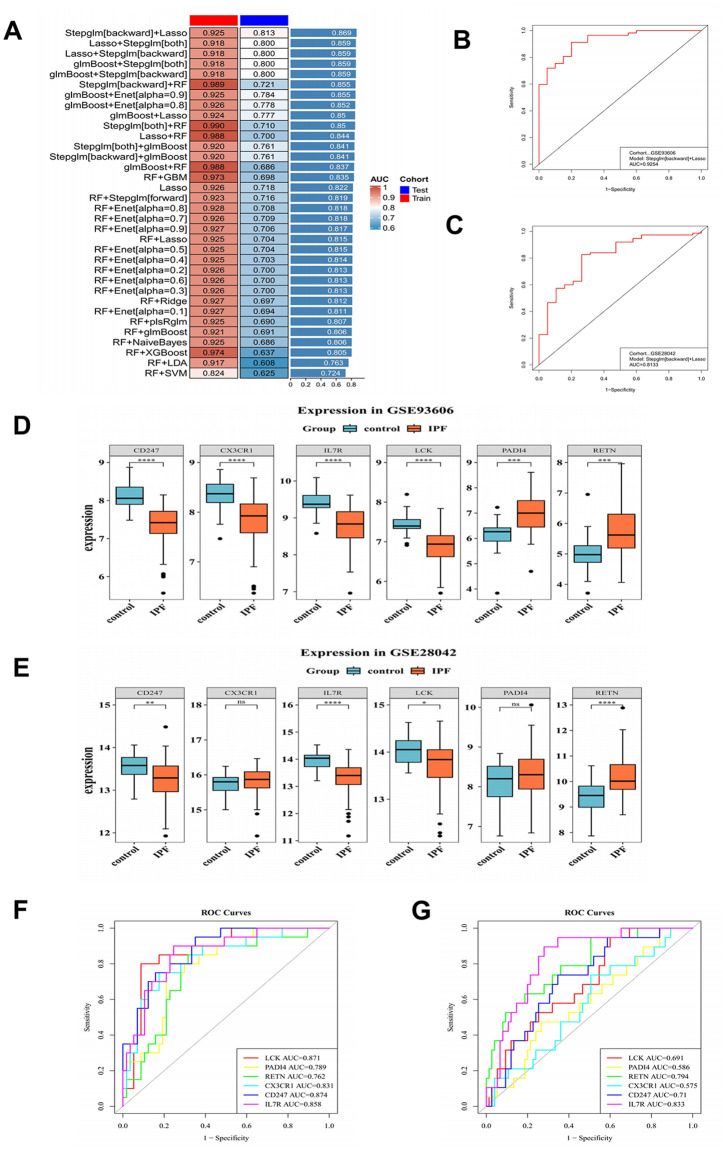
Identification of *CD247, RETN,* and *IL7R* as biomarkers using a comprehensive analysis of 101 machine-learning models. (A) AUC values of the models on the training and validation sets. (B) and (C) ROC curves of the optimal model in the training and validation sets, respectively. (D) and (E) Boxplots of feature gene expression levels in the training and validation sets, respectively. Red represents IPF samples, and blue represents control samples. (F) and (G) ROC curves of feature genes in the training and validation sets, respectively.

Biomarkers were identified through machine-learning analysis, gene expression profiling, and diagnostic performance evaluation. The optimal composite model was Stepglm[backward] + LASSO, which satisfied the predefined variable-count and AUC criteria in both datasets (Fig 2A-C). Specifically, the AUC values of this model were 0.9254 in GSE93606 and 0.8133 in GSE28042. This model included six feature genes: *LCK, PADI4, RETN, CX3CR1, CD247,* and *IL7R*. Expression analysis showed that *CD247, IL7R, LCK,* and *RETN* exhibited consistent expression trends across both datasets (Fig 2D-E). Notably, *RETN* was upregulated in IPF, whereas *CD247, IL7R,* and *LCK* were downregulated (p < 0.05). ROC analysis in GSE93606 yielded AUC values of 0.874 for *CD247*, 0.762 for *RETN*, and 0.858 for *IL7R* (Fig 2F). Similarly, in GSE28042, the corresponding AUC values were 0.710, 0.794, and 0.833, respectively (Fig 2G). These findings suggest that these genes have diagnostic value. In summary, *CD247, RETN,* and *IL7R* showed consistent expression patterns across both datasets and demonstrated diagnostic value, supporting their potential as candidate biomarkers.

### The predictive performance of *CD247, RETN,* and *IL7R* in IPF

**Fig 3:**
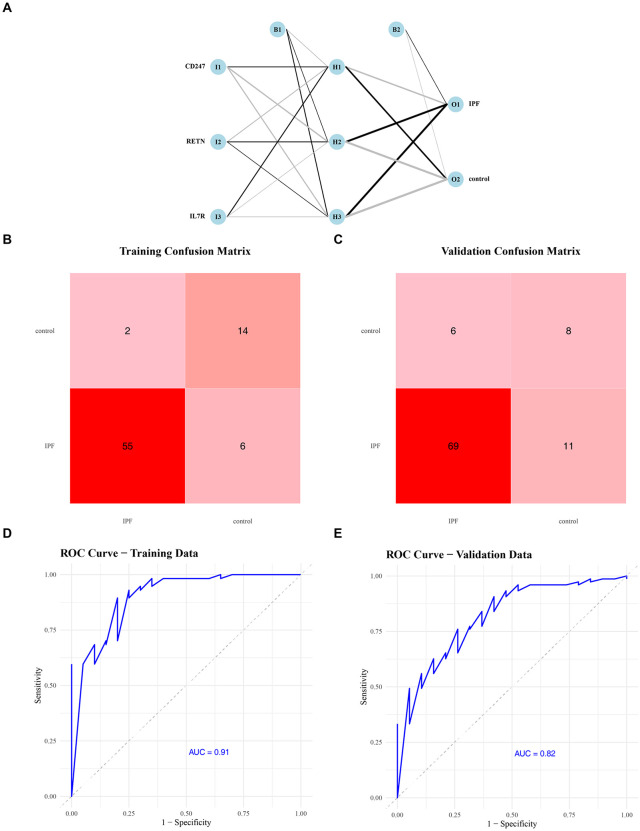
Predictive performance of *CD247, RETN,* and *IL7R* in IPF. (A) Architecture of the artificial neural network (ANN) model. Nodes represent neurons, and edges represent weighted connections; color intensity reflects connection strength. (B–C) Confusion matrices for the training and validation cohorts. (D–E) Receiver operating characteristic (ROC) curves for the training and validation cohorts. The ANN achieved an AUC of 0.91 in the training set and 0.82 in the validation set.

To evaluate the predictive performance of *CD247, RETN,* and *IL7R* in idiopathic pulmonary fibrosis (IPF), an artificial neural network (ANN) model was constructed (Fig 3A). Confusion matrix analyses demonstrated strong classification performance in both the training and validation cohorts (Fig 3B–C). In the training dataset (GSE93606), the model achieved an area under the ROC curve (AUC) of 0.91, while in the independent validation dataset (GSE28042), the AUC remained at 0.82 (Fig 3D–E), indicating good discriminative ability across datasets. Detailed performance metrics further supported the robustness of the model (Table 2). In the training cohort (n = 77), accuracy, precision, recall (sensitivity), and F1 score were 0.883, 0.900, 0.947, and 0.923, respectively. In the validation cohort (n = 94), these metrics remained consistently high, with accuracy of 0.851, precision of 0.877, recall of 0.947, and F1 score of 0.910. Collectively, these results indicate that the ANN model based on the identified biomarkers effectively distinguishes IPF from control samples and exhibits encouraging discriminative performance in the analyzed datasets, while further validation will be necessary to determine its broader reproducibility and clinical applicability.

**Table 2:** Performance metrics derived from the confusion matrices.

Group	TP	TN	FP	FN	Accuracy	Precision	Recall	F1_Score
GSE93606	54	14	6	3	0.8831	0.9	0.9474	0.9231
GSE28042	71	9	10	4	0.8511	0.8765	0.9467	0.9103

### Analysis of chromosomal localization and functional assessment of *CD247, RETN,* and *IL7R*

**Fig 4:**
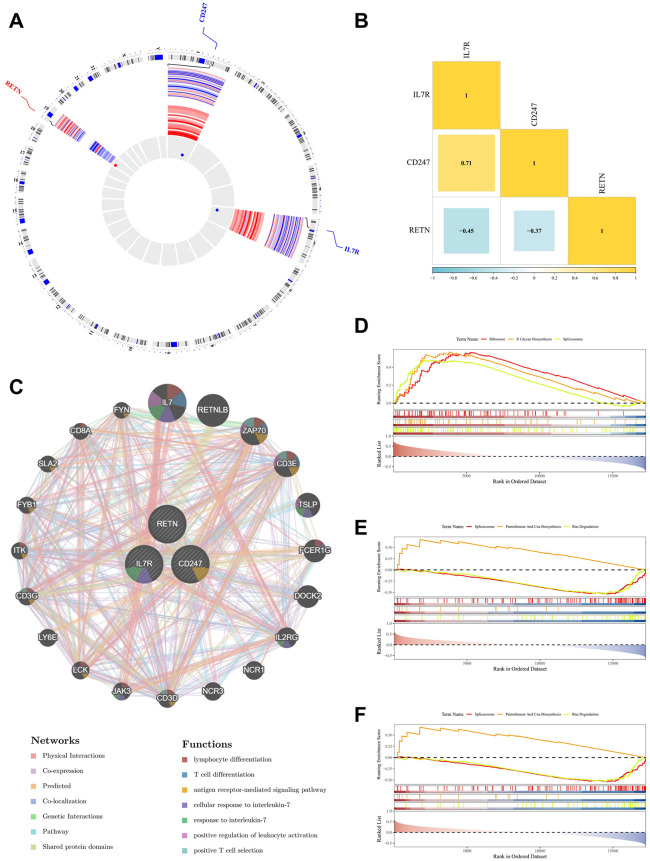
Analysis of chromosomal localization and functional assessment of *CD247, RETN,* and *IL7R.* (A) Chromosomal localization map. (B) Correlation heatmap of biomarkers. (C) GeneMANIA network of biomarkers. The circles in the figure represent co-expressed genes of the biomarkers, and different colored blocks reflect their involved functions. (D), (E), and (F) Top three GSEA-enriched pathways for *CD247, RETN,* and *IL7R*, respectively.

Chromosomal mapping was performed to visualize the genomic distribution of the identified biomarkers. The results showed that *CD247* is located on chromosome 1, *IL7R* on chromosome 5, and *RETN* on chromosome 19 (Fig 4A). Correlation analysis showed that these biomarkers were significantly interrelated (|cor| > 0.3, p < 0.05) (S5 Table), with a particularly strong positive correlation observed between *CD247* and *IL7R* (cor = 0.71, p < 0.05) (Fig 4B). To further explore their functional context, interactions among the biomarkers and their related genes were examined. The GeneMANIA analysis showed that co-expressed genes associated with these biomarkers included *IL7, RETNLB, ZAP70, CD3E,* and *TSLP,* which were primarily involved in functions such as lymphocyte differentiation, T cell differentiation, the antigen receptor-mediated signaling pathway, and the cellular response to interleukin-7 (Fig 4C). GSEA revealed that *CD247* was associated with 30 pathways, *RETN* with 36 pathways, and *IL7R* with 50 pathways (adjusted p-value < 0.05). Notably, *CD247* was primarily enriched in the ribosome, N-glycan biosynthesis, and spliceosome pathways (Fig 4D). For *IL7R*, the leading pathways were ribosome, spliceosome, and N-glycan biosynthesis (Fig 4E). The top three pathways enriched for *RETN* included spliceosome, pantothenate and CoA biosynthesis, and RNA degradation (Fig 4F). Notably, the spliceosome pathway was among the top three pathways enriched for all three genes.

### Immune infiltration analysis of *CD247, RETN,* and *IL7R*

**Fig 5:**
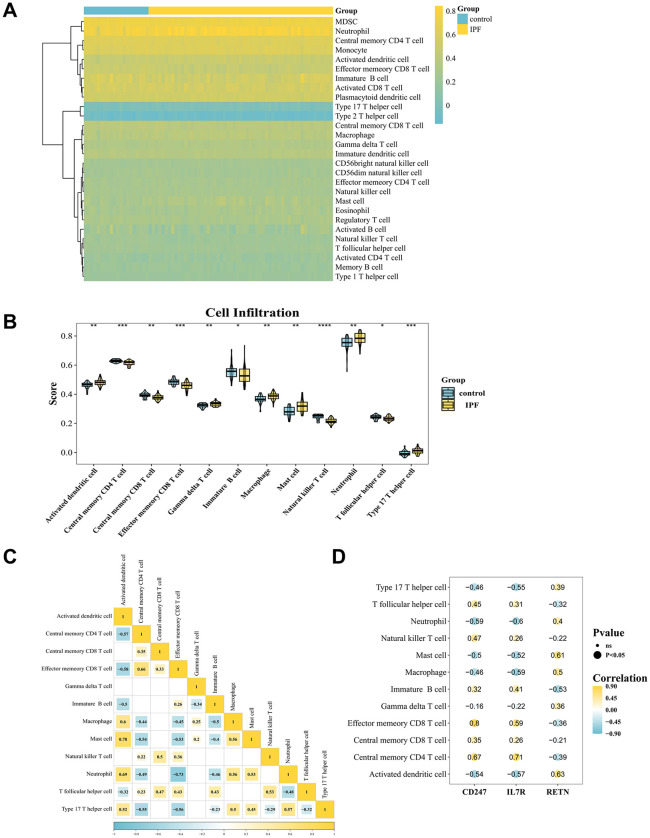
Immune infiltration analysis of *CD247, RETN,* and *IL7R.* (A) Heatmap of immune cell enrichment scores in the IPF and control groups. The color represents the enrichment score, with yellow indicating a score greater than 0 and blue representing a score less than 0. The darker the color, the higher the score. (B) Differences in immune cell enrichment scores between IPF patients and control samples. Yellow represents IPF samples, and blue represents control samples. (C) Correlation heatmap of differential immune cell populations. Blue represents negative correlation, yellow represents positive correlation, and the darker the color, the greater the correlation. Blank areas in the figure represent insignificant p-values. (D) Correlations between differential immune cell populations and biomarkers. Blue represents negative correlation, yellow represents positive correlation, and the darker the color, the greater the correlation. The size of the circle indicates whether the p-value is significant, with numbers on the circles representing the magnitude of the correlation.

In GSE93606, the extent of infiltration of 28 immune cell types was assessed in IPF and control samples (Fig 5A, S6 Table). Analysis of immune cell infiltration revealed significant differences in the enrichment scores of 12 cell types (p < 0.05), including natural killer T (NKT) cells, CD4 central memory T cells, and neutrophils, between the IPF and control groups (Fig 5B). Among these differential immune cell populations, mast cells and activated dendritic cells showed the strongest positive correlation (cor = 0.78, p < 0.05), whereas neutrophils and CD8 memory effector T cells showed the strongest negative correlation (cor = −0.73, p < 0.05) (Fig 5C). Analysis of biomarker–immune cell associations further showed a strong positive correlation between *CD247* and CD8 memory effector T cells (cor = 0.80, p < 0.05), whereas *IL7R* showed the strongest negative correlation with neutrophils (cor = −0.60, p < 0.05) (Fig 5D).

### Regulatory network analysis of *CD247, RETN,* and *IL7R*

**Fig 6:**
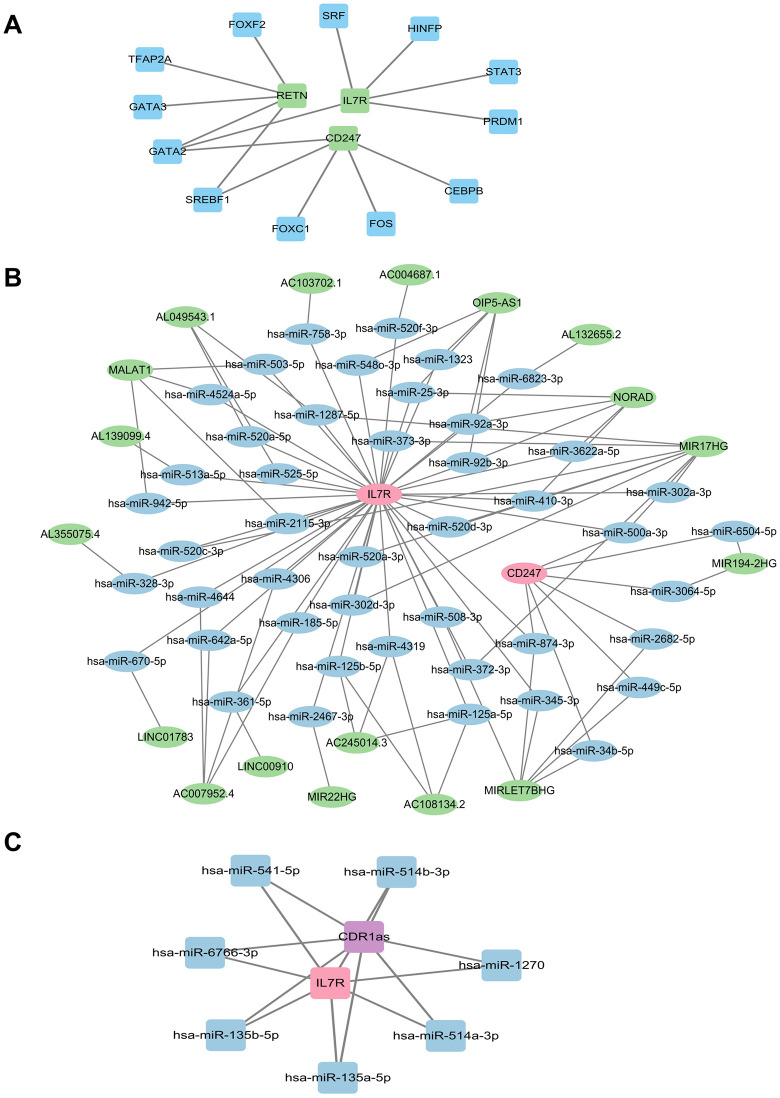
Regulatory network analysis of *CD247, RETN,* and *IL7R.* (A) Transcription factor (TF)–mRNA regulatory network of the biomarkers. Green nodes represent biomarkers, and blue nodes represent TF. (B) lncRNA–miRNA–mRNA regulatory network of the biomarkers. Pink nodes represent genes, blue nodes represent miRNAs, and green nodes represent lncRNAs. (C) circRNA–miRNA–mRNA regulatory network of the biomarkers. Pink nodes represent genes, blue nodes represent miRNAs, and purple nodes represent circRNAs.

The transcription factor (TF)–mRNA regulatory network suggested that *CD247*, *IL7R*, and *RETN* were potentially regulated by a total of 12 transcription factors based on the JASPAR database, among which GATA2 was identified as a shared regulator (Fig 6A). In the miRWalk database, *CD247*, *RETN*, and *IL7R* were associated with 1,579, 1,042, and 1,940 miRNAs, respectively. In the TargetScan database, *CD247*, *RETN*, and *IL7R* were linked to 350, 21, and 1,042 miRNAs, respectively. Overlap analysis across the two databases identified 250 shared miRNAs for *CD247*, 12 for *RETN*, and 713 for *IL7R* (S7 Table). Subsequently, 18 lncRNAs were predicted using the StarBase database, whereas no lncRNAs were identified for *RETN*. Based on these results, an lncRNA–miRNA–mRNA regulatory network was constructed (Fig 6B and S8 Table). Finally, using the intersecting miRNAs, one circRNA (*CDR1as*) was predicted in the StarBase database, and a circRNA–miRNA–mRNA regulatory network was established (Fig 6C).

### Drug prediction and gene-drug binding profiling

**Fig 7:**
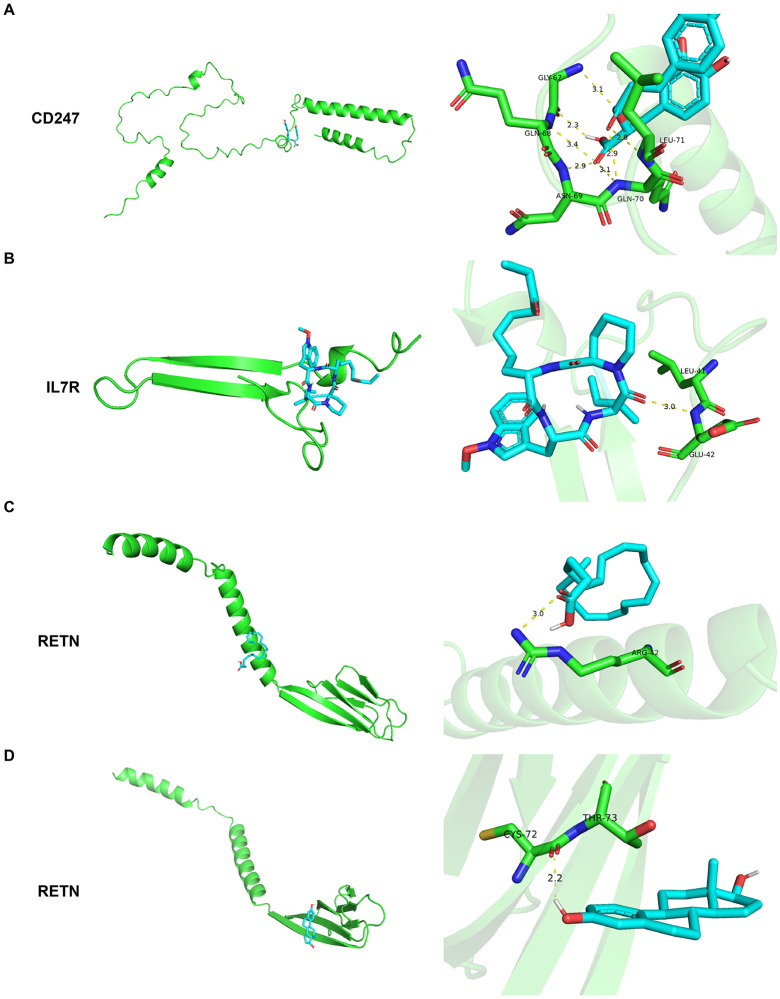
Drug prediction and molecular docking profiles. Molecular docking diagrams for CD247 (A), IL7R (B), and RETN (C-D) are shown from top to bottom. The stick models represent the molecular structures of the active compounds, with labeled amino acid residues indicating the docking sites. The left panels display the overall docking views, while the right panels provide magnified local views.

The analysis identified two compounds associated with RETN, one compound linked to IL7R, and one compound related to CD247 (Table 3). Molecular docking analysis indicated that the predicted compounds formed hydrogen-bond interactions within functional domains of their respective target proteins. CD247 showed binding affinity with romoxacin (Vina score = –6.2 kcal/mol), involving residues GLY-67, GLN-68, ASN-69, GLN-70, and LEU-71 (Fig 7A). IL7R bound apicidin with a Vina score of –6.2 kcal/mol, forming interactions at residues LEU-41 and GLU-42 within the IL-7 binding domain (Fig 7B). RETN exhibited binding affinities with arachidonic acid (–4.1 kcal/mol) and estradiol (–5.2 kcal/mol), interacting at residues ARG-42, CYS-72, and THR-73 (Fig 7C–D). All binding sites were located within known functional domains; however, the potential functional relevance of these interactions remains speculative and requires experimental validation.

**Table 3:** Drug prediction and molecular docking profiling.

Genes	AlphaFold ID	Ingredients	PubChem CID	Vina score (kcal/mol)	Residues	Hydrogen bond distance
RETN	AF-Q9HD89-F1-v4	arachidonic acid	444899	−4.1	ARG-42	3.0Å
RETN	AF-Q9HD89-F1-v4	estradiol	5757	−5.2	CYS-72 THR-73	2.2Å
IL7R	AF-F6IQ79-F1-v4	apicidin	6918328	−6.2	LEU-41 GLU-42	3.0Å
CD247	AF-P20963-F1-v4	rosmarinic acid	5281792	−6.2	GLY-67 GLN-68 ASN-69 GLN-70 LEU-71	2.3Å 2.8Å 2.9Å 3.1Å 3.4Å

### Annotation of 4 major cell types and identification of monocytes as the predominant biomarker-associated cell population

**Fig 8:**
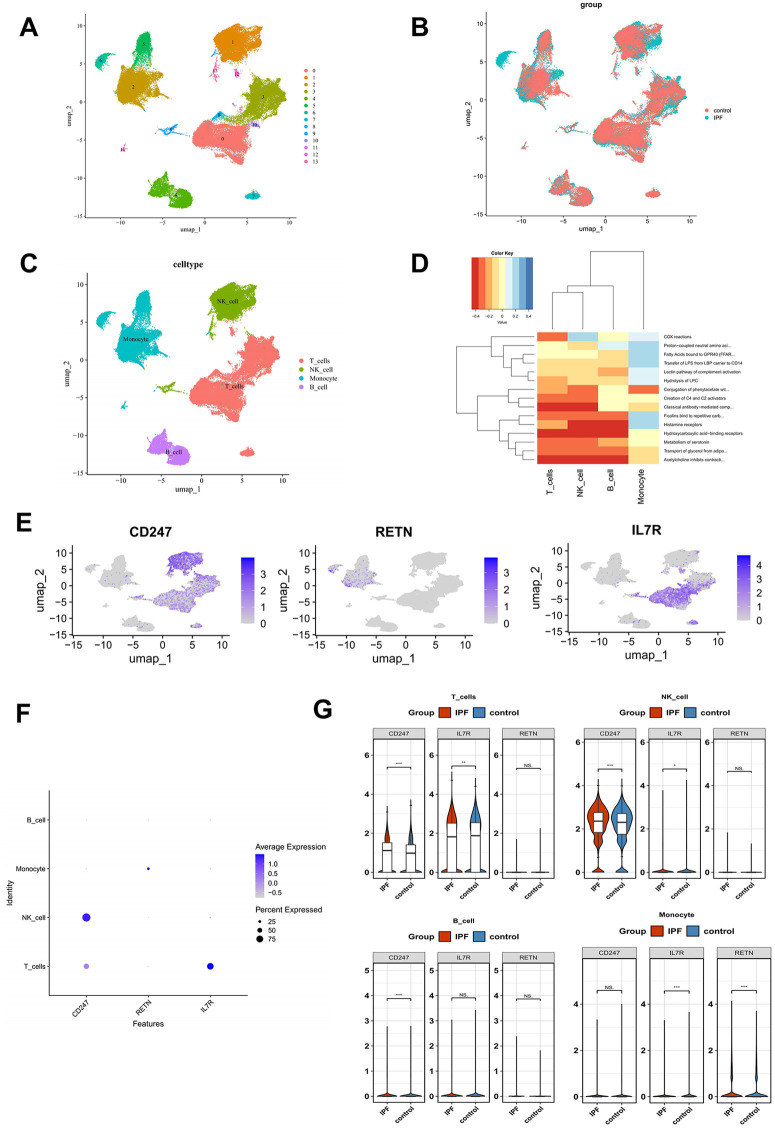
Annotation of four major immune cell types and identification of monocytes as the predominant biomarker-associated cell population. (A) UMAP plot of cell clustering. (B) Cell clustering plot of IPF and control samples. (C) Cell type annotation. (D) Heatmap of functional enrichment analysis. Colors represent enrichment scores. (E) UMAP plot of biomarker expression levels. (F) Expression of biomarkers across different cell types. Cell types are ordered by the average expression values. (G) Violin plot of biomarker expression levels.

All scRNA-seq–based analyses, including cell-type annotation, biomarker expression profiling, and pseudotime trajectory inference, were performed using a single publicly available IPF scRNA-seq dataset (GSE233844). After preprocessing and quality control, a total of 121,210 cells and 19,900 genes met the inclusion criteria. Initially, we generated visualizations of nFeature RNA, nCount RNA, and the percentage of mitochondrial content before and after quality control (S1A-B Fig). For downstream analyses, 2,000 HVGs were selected, and the 10 HVGs with the largest variation were labeled (S1C Fig). After PCA, the top 30 principal components were selected for further analysis (S1D-E Fig). Clustering analysis identified 14 distinct cell populations (Fig 8A-B), which were further annotated into four major immune cell types: T cells, natural killer (NK) cells, monocytes, and B cells (Fig 8C). Functional enrichment analysis was conducted on these four cell types, identifying 1,795 differentially enriched pathways; the heatmap shows the top 15 most significantly enriched pathways (e.g., histamine receptors and COX reactions) (Fig 8D) (p < 0.05). Notably, *CD247* exhibited higher expression levels in T cells and NK cells, while *RETN* was predominantly expressed in monocytes, and *IL7R* showed elevated levels in T cells (Fig 8E-F). These four cell types were subsequently used to compare biomarker expression between IPF and control samples. In T cells and NK cells, *CD247* and *IL7R* displayed differential expression between the IPF and control groups; in monocytes, *IL7R* and *RETN* showed differential expression, and in B cells, *CD247* was differentially expressed (Fig 8G). Monocytes showed the most prominent biomarker-associated differential expression among the annotated cell populations (p < 0.001) in this dataset and were therefore selected for downstream exploratory analyses.

### Cell communications and pseudo-time trajectory inference of monocytes

**Fig 9:**
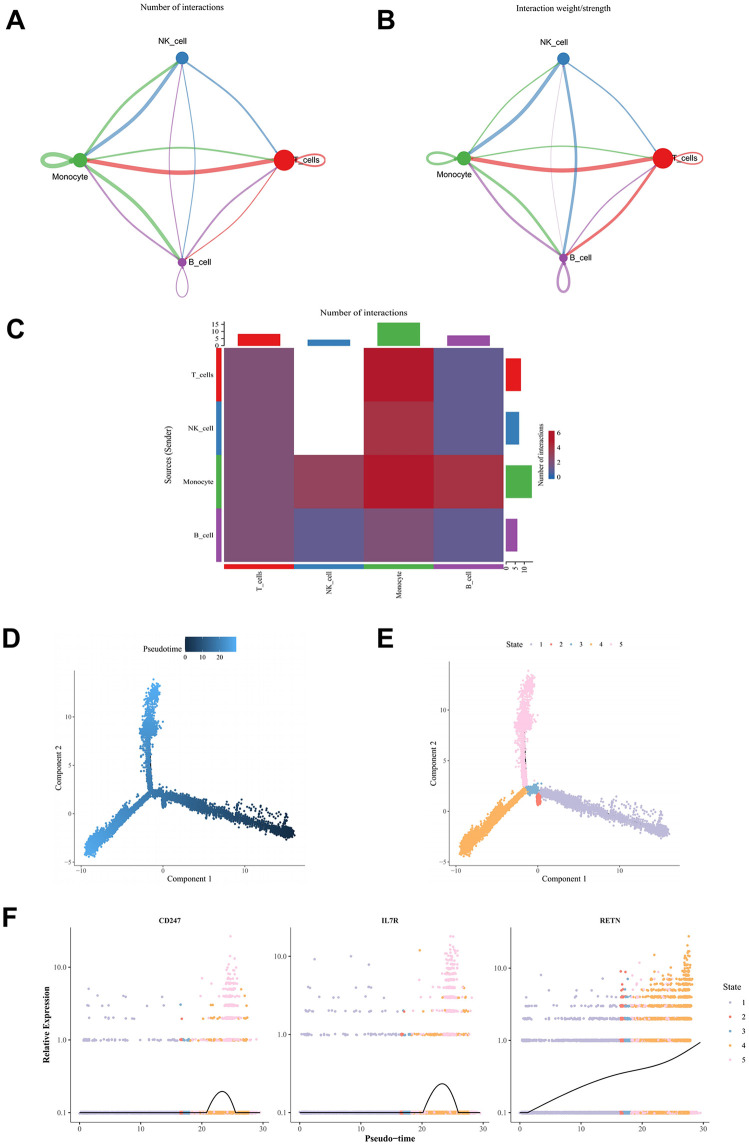
Cellular communication and pseudotime trajectory analysis. (A) Interaction count network between monocytes and other cell types. The nodes represent different cell types, and the size of the nodes indicates the number of cells in each type. The thickness of the lines represents the number of communications. (B) Interaction strength network between monocytes and other cell types. The nodes represent different cell types, and the size of the nodes indicates the number of cells in each type. The thickness of the lines represents the strength of communications. (C) Interaction diagram of ligand-receptor pairs between cell types. The vertical axis represents the signaling cells, and the horizontal axis represents the receiving cells. The color intensity of the heatmap indicates the strength of the signal. The bars on the top and right side represent the cumulative counts of the vertical and horizontal axes, respectively. (D) Pseudotime trajectory of cell differentiation. Dark blue represents the early stages of differentiation, while light blue represents the later stages. (E) Different states of cells. (F) Expression trends of biomarkers during cell differentiation.

Analysis of communication among annotated cell types provided further insight into intercellular interactions. The frequency and intensity of interactions indicated that monocytes and T cells engaged more frequently and with greater strength (Fig 9A-C). Trajectory analysis revealed that the expression levels of *CD247, RETN,* and *IL7R* fluctuated over differentiation time. Specifically, *CD247* and *IL7R* exhibited an initial increase followed by a decrease, while *RETN* showed a gradual increase over time (Fig 9D-F).

### Experimental validation of *CD247, IL7R*, and *RETN*

**Fig 10:**
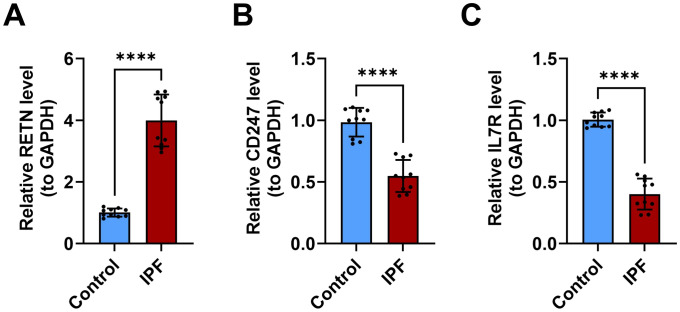
Validation of biomarker expression levels in peripheral blood samples from 10 patients with IPF and 10 healthy controls. (A) The mRNA expression level of *RETN*. (B) The mRNA expression level of *CD247*. (C) The mRNA expression level of *IL7R*. Note: **p < 0.01, ***p < 0.001.

RT-qPCR validation showed that, compared with healthy controls, *RETN* mRNA expression was significantly increased in peripheral blood samples from patients with IPF, whereas *CD247* and *IL7R* mRNA expression levels were significantly decreased (Fig 10A–C). These results provided preliminary experimental support for the differential expression of these biomarkers in IPF.

## Discussion

Idiopathic pulmonary fibrosis (IPF) is a progressive fibrotic lung disease in which mitochondrial dysfunction is increasingly implicated in pathogenesis. By integrating bulk transcriptomics, machine-learning pipelines, and single-cell analyses, this study identified candidate biomarkers related to mitochondrial dynamics-associated pathways and characterized their cellular distribution in IPF. The principal finding is that *CD247*, *IL7R*, and *RETN* discriminate IPF from controls with single-gene AUCs > 0.7 and collectively support a neural-network classifier with encouraging preliminary performance (training AUC 0.91; validation AUC 0.82). Single-cell analyses based on one publicly available scRNA-seq dataset suggested that their dysregulation was most evident in monocyte-associated compartments and revealed stage-associated expression dynamics. These observations support a potential link between immune dysregulation and altered mitochondrial dynamics in IPF, although causal relationships remain to be established.

*CD247* encodes the CD3ζ chain, a core component of the T-cell receptor complex [27]. Reduced *CD247* expression has previously been associated with impaired gas exchange and prognosis in IPF [28], and epigenetic downregulation has also been reported in COPD [29]. Consistent with these observations, *CD247* was decreased across IPF cohorts in the present study. Because T-cell activation is closely coupled to mitochondrial metabolic reprogramming, reduced *CD247* expression may reflect altered TCR-associated immune-metabolic states, although this interpretation remains indirect.

*IL7R* encodes the interleukin-7 receptor α chain and is essential for lymphocyte survival and development [30,31]. In addition to its developmental role, IL-7/*IL7R* signaling has been linked to redox balance and immune regulation [32–34]. We observed consistent downregulation of *IL7R* across IPF datasets. Given prior evidence that IL-7 signaling influences mitochondrial integrity and apoptotic thresholds in immune cells, reduced *IL7R* expression may be associated with altered mitochondrial homeostasis and impaired adaptive immune regulation, although this remains inferential.

*RETN* encodes resistin, a cytokine involved in inflammatory and metabolic regulation [35–37]. Although direct evidence in IPF remains limited, *RETN* was reproducibly upregulated and showed stable diagnostic performance across independent datasets. Resistin has been associated with oxidative stress and macrophage inflammatory activation, processes often accompanied by mitochondrial perturbation. The progressive increase of *RETN* along monocyte pseudotime trajectories is consistent with enhanced innate immune activation, but current evidence supports *RETN* primarily as an inflammatory-associated biomarker rather than a confirmed mechanistic driver of fibrosis.

Taken together, *CD247*, *IL7R*, and *RETN* may represent complementary components of a shared immune-mitochondrial axis. *CD247* and *IL7R* were more closely associated with adaptive immune signaling, whereas *RETN* was more strongly linked to innate inflammatory activation, particularly within monocytes. This partial functional complementarity may help explain the superior performance of the multi-marker ANN model relative to single-gene classifiers and supports further evaluation of these markers in future biomarker-validation and model-development studies.

Pathway analyses revealed convergence on RNA splicing–related processes, consistent with reports implicating spliceosome components in fibrosis [38]. Although the identified biomarkers are not core spliceosome constituents, their enrichment in splicing-associated gene sets suggests that immune activation and mitochondrial perturbation may coincide with altered RNA-processing programs in IPF. Aberrant alternative splicing has been increasingly recognized in pulmonary fibrosis, influencing epithelial function, fibroblast activation, and immune signaling. These observations are hypothesis-generating and warrant future studies evaluating whether mitochondrial stress and immune dysregulation are accompanied by measurable changes in splicing factor activity or transcript isoform composition.

Mitochondrial dynamics regulate immune-cell activation and differentiation [39]. Key regulators such as DRP1 and MFN1/2 influence macrophage and T-cell programs [40,41], and altered dynamics can reshape cellular bioenergetics and signaling [42]. Increased fission has been linked to effector phenotypes, whereas fusion favors antigen-presenting states [43,44]. Consistent with this framework, immune-infiltration analysis (Results 3.5) revealed significant differences in multiple immune-cell populations in IPF (p < 0.05). *CD247* correlated positively with CD8 effector-memory T cells and negatively with neutrophils, and *IL7R* also showed a negative association with neutrophils. Given reports that neutrophil accumulation contributes to tissue injury and fibrotic remodeling in IPF [45–47], these findings are compatible with a relative shift toward innate inflammatory predominance. However, these associations are correlative and do not establish causality.

Established serum biomarkers—including KL-6, MMP-7, SP-A/SP-D, and periostin—have shown moderate diagnostic utility in IPF [48]. In comparison, the single-gene AUCs (> 0.7) for *CD247*, *IL7R*, and *RETN*, together with the improved performance of the multi-marker ANN model, appear comparable to reported markers. However, these comparisons should be interpreted cautiously because the current study was not designed as a head-to-head clinical validation analysis. Notably, whereas conventional biomarkers primarily reflect epithelial injury or fibrotic burden, the markers identified here are associated with immune regulation and mitochondrial dynamics, suggesting potential complementarity in future multi-marker validation frameworks rather than redundancy.

Regulatory-network analyses integrating transcription factors and non-coding RNAs (lncRNAs/miRNAs/circRNAs) identified computationally inferred upstream regulators of *CD247*, *IL7R*, and *RETN*. Among circRNAs, *CDR1as* has been linked to TGF-β–dependent invasive and epithelial–mesenchymal transition (EMT)–like programs across disease contexts [49–53]. Given the established role of TGF-β signaling in pulmonary fibrogenesis and its interaction with mitochondrial metabolism, circRNA–miRNA–TGF-β circuits may represent an inferred regulatory layer potentially related to immune–mitochondrial imbalance in IPF.

Although these interactions were inferred computationally, several predicted regulators have prior relevance to fibrotic and immune processes. *GATA2*, predicted to regulate *CD247*, *IL7R*, and *RETN*, participates in immune-cell differentiation and tissue repair, while lnc-*IL7R* modulates inflammatory signaling and oxidative stress responses in chronic lung disease [54]. These findings support the biological plausibility of the inferred networks but require experimental validation.

Together, these prior findings support the biological plausibility of the predicted TF–ncRNA–mRNA regulatory networks; however, these inferred regulatory relationships should be regarded as hypothesis-generating and do not constitute direct evidence of validated molecular regulation or translational relevance. Future studies should prioritize experimental confirmation of these regulatory interactions, including validation of upstream immune signaling pathways, assessment of neutrophil-associated inflammatory programs, and mechanistic dissection of circRNA–miRNA–TGF-β circuits in relevant cellular and tissue contexts.

Single-cell analyses (Results 3.8–3.9) in one publicly available dataset highlighted monocytes as candidate cellular mediators warranting further validation. Monocyte recruitment and differentiation have been implicated in fibrotic progression, including radiation-induced lung injury [55,56]. Pseudotime trajectories revealed early up-then-down patterns for *CD247* and *IL7R* and progressive increases for *RETN*, patterns that may be consistent with transitions from early immune activation and survival signaling toward later inflammatory–fibrotic programs [27,57,58].

Accumulating evidence underscores the role of monocytes and their derivatives in IPF pathogenesis. Monocyte-derived alveolar macrophages correlate with fibrotic severity and actively promote lung injury and fibrosis in human and experimental settings [59]. Increased circulating monocytes and dysregulated monocyte gene programs have also been associated with adverse outcomes in IPF [60], and single-cell transcriptomic studies have identified aberrant monocyte phenotypes characterized by elevated CD64 expression and enhanced type I interferon signaling [61]. In this context, the prominence of monocyte-associated expression patterns for *CD247, IL7R,* and *RETN* in this dataset is consistent with emerging immunopathological models.

By incorporating pseudotime analysis, we extended beyond static differential expression and captured dynamic regulation of candidate biomarkers related to mitochondrial dynamics-associated pathways during monocyte differentiation. *CD247* and *IL7R* displayed early upregulation followed by gradual decline, whereas *RETN* increased progressively, suggesting temporally distinct expression patterns that may reflect evolving immune states along differentiation trajectories. While these patterns do not establish causality, they provide a temporal framework for future mechanistic investigation. However, because single-cell transcriptomics remains vulnerable to dataset-specific batch effects, annotation uncertainty, and the absence of orthogonal protein-level or spatial validation, these findings should be interpreted as exploratory and will require confirmation in independent cohorts and with complementary experimental approaches.

Molecular docking analyses in this study should be interpreted as exploratory computational predictions rather than evidence of direct biological activity or therapeutic relevance. Because the docking workflow was based on predicted protein structures and relative binding energies, the identified ligand–target pairs should be regarded as hypothesis-generating candidates only. Accordingly, CD247, IL7R, and RETN cannot be considered validated pharmacologic targets on the basis of these results alone, and the candidate ligands identified here should not be interpreted as confirmed therapeutic agents. Dedicated experimental validation, including biophysical binding assays, functional cellular studies, and target-specific perturbation experiments, will be required before any translational conclusions can be drawn.

In summary, *CD247, IL7R*, and *RETN* are associated with immune regulation, mitochondrial dynamics, and RNA-processing pathways in IPF. Although direct functional validation was beyond the scope of this study, the integrative transcriptomic and single-cell analyses should be interpreted as exploratory and hypothesis-generating, and they provide testable hypotheses regarding immune–mitochondrial interactions in fibrotic progression. These relationships remain inferential and will require perturbational studies, such as gene knockdown, overexpression, or CRISPR-based modulation, to clarify causal roles. Accordingly, future studies should prioritize independent cohort replication, improved annotation robustness, orthogonal validation, and functional perturbation experiments to establish the reproducibility and biological significance of these observations.

## Conclusions

By integrating bulk and single-cell transcriptomics with multiple machine-learning approaches, we identified *CD247, IL7R*, and *RETN* as candidate biomarkers related to mitochondrial dynamics-associated pathways in IPF and provided preliminary external support through RT-qPCR validation. Each marker demonstrated AUC > 0.7 across independent datasets, and their combination supported a high-performing ANN classifier. Enrichment and network analyses linked these markers to immune and RNA-splicing pathways, while single-cell mapping in one publicly available dataset suggested that dysregulation was most evident in monocyte-associated compartments. These findings support future biomarker-validation and multi-marker model-development efforts and suggest that immune–mitochondrial interactions may represent an area for further mechanistic investigation.

## Limitations and future perspectives

This study has several limitations. First, analyses were based largely on public transcriptomic datasets and computational modeling; therefore, batch effects, cohort heterogeneity, and algorithmic bias may influence results, and the observed associations cannot establish causality. Second, RT-qPCR validation was performed in a relatively small cohort of 10 patients with idiopathic pulmonary fibrosis and 10 healthy controls. Although this validation supported the overall direction of biomarker expression changes, the modest sample size may have limited statistical power and reduced the generalizability of the findings. Therefore, these results should be interpreted as preliminary experimental support rather than definitive validation. Third, single-cell analyses were derived from a single publicly available dataset and performed using a low clustering resolution designed to prioritize major immune populations rather than fine-grained subclusters. Therefore, the observed monocyte-associated expression patterns and pseudotime dynamics should be considered exploratory. In addition, single-cell transcriptomic studies remain vulnerable to batch effects, annotation uncertainty, and limited capacity for direct functional or protein-level confirmation. Finally, functional roles of *CD247, IL7R*, and *RETN* were not directly tested, and the predicted regulatory networks and ligand–target interactions were not experimentally validated. Taken together, these limitations indicate that the current findings should be interpreted as preliminary, integrative, and hypothesis-generating rather than as definitive mechanistic or clinically actionable evidence.

Future work should include larger multicenter validation cohorts, protein-level and spatial confirmation of cell-type-specific expression, and perturbational studies to assess whether modulation of *CD247*, *IL7R*, or *RETN* influences fibrogenic responses. Experimental validation of predicted regulatory networks and drug-target interactions will also be necessary, including functional cellular studies and targeted validation approaches. These efforts may clarify the biological and translational relevance of the identified biomarkers in IPF. Future studies should also prioritize independent cohort replication, cross-dataset harmonization when multiple scRNA-seq datasets are available, improved annotation robustness, and orthogonal validation using approaches such as flow cytometry, immunohistochemistry, immunofluorescence, protein quantification, and spatial transcriptomics.

## Supporting information

S1 Fig Single-cell analysis. (A) Distribution plots of nFeature_RNA and nCount_RNA before quality control. (B) Distribution plots of nFeature_RNA and nCount_RNA after quality control. (C) Identification of highly variable genes. Red dots represent variable genes, whereas black dots represent non-variable genes. (D) PCA scatter plot of highly variable genes. (E) JackStraw plot for PCA-based dimensionality reduction of highly variable genes.
(TIF)

S1 Table Differentially expressed genes (DEGs). (XLSX)

S2 Table Results of consensus clustering analysis. (XLSX)

S3 Table GO enrichment analysis of candidate genes. (XLSX)

S4 Table KEGG pathway enrichment analysis of candidate genes. (XLSX)

S5 Table Correlation analysis of biomarkers. (XLSX)

S6 Table Correlation analysis among infiltrating immune cell types in the GSE93606 dataset. (XLSX)

S7 Table Statistical analysis of the intersection of targeted gene miRNAs predicted by different databases. (XLSX)

S8 Table Detailed information for the lncRNA–miRNA–mRNA regulatory network. (XLSX)
