# Supplementary figures and images for: Biomarkers of mitochondrial dynamics in idiopathic pulmonary fibrosis: Identification and validation through transcriptomic and single-cell analyses
Source: PLoS One. 2026 Apr 23;21(4):e0347845. doi: 10.1371/journal.pone.0347845 (PMC13105346; doi:10.1371/journal.pone.0347845)

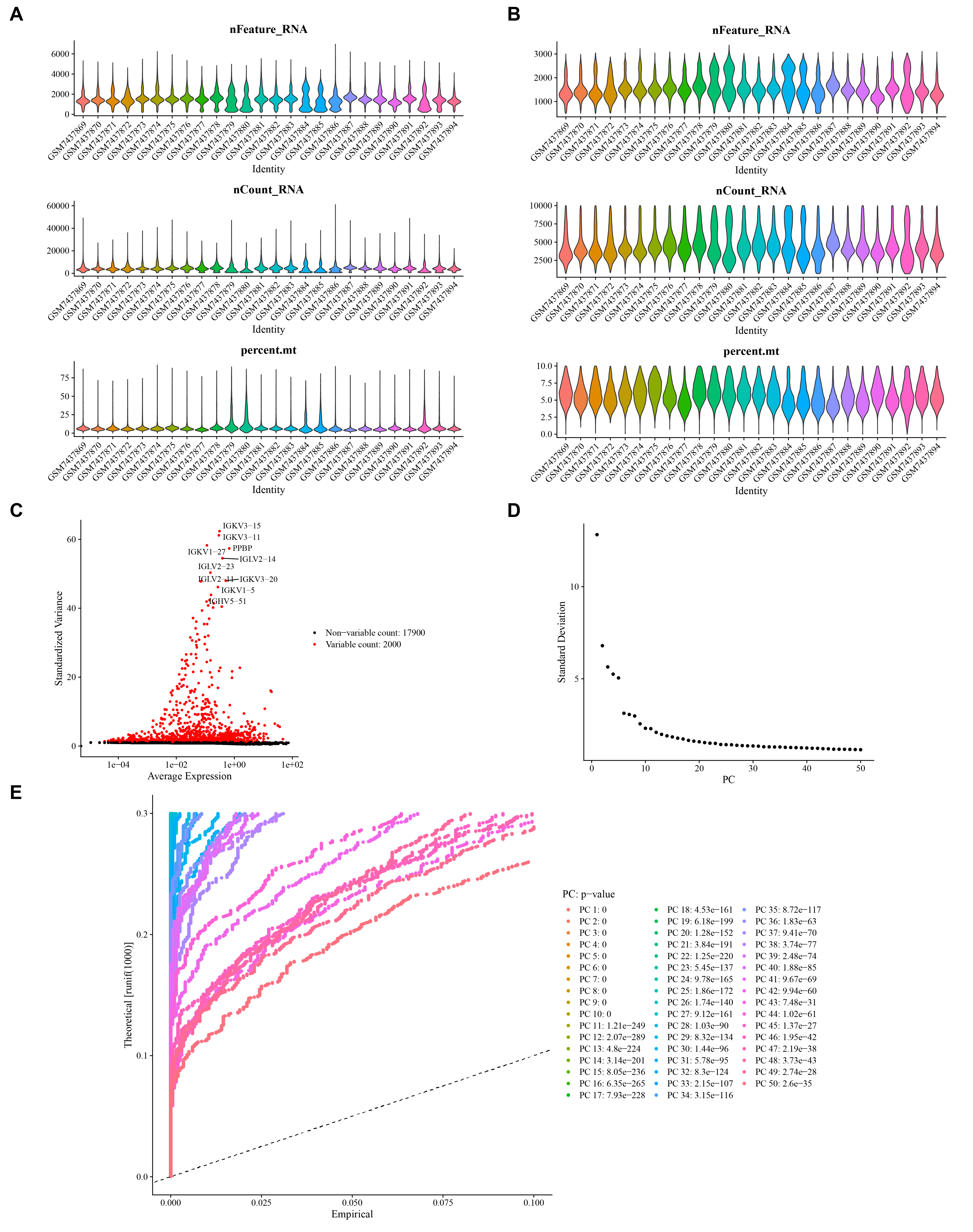

Supplement: S1 Fig — (A) Distribution plots of nFeature_RNA and nCount_RNA before quality control. (B) Distribution plots of nFeature_RNA and nCount_RNA after quality control. (C) Identification of highly variable genes. Red dots represent variable genes, whereas black dots represent non-variable genes. (D) PCA scatter plot of highly variable genes. (E) JackStraw plot for PCA-based dimensionality reduction of highly variable genes. (TIF) [file pone.0347845.s001.tif]
